# Niraparib Plus Aromatase Inhibitors for Hormone Receptor-Positive/HER2-Negative Advanced Breast Cancer with a Germline *BRCA* Mutation

**DOI:** 10.3390/cancers17111744

**Published:** 2025-05-22

**Authors:** Laura Lema, José Manuel Pérez-García, Salvador Blanch, Judith Balmaña, José Ángel García-Sáenz, Elena Filipovich Vegas, Begoña Jiménez, Juan de la Haba, Marta Campolier, Eileen Shimizu, Daniel Alcalá-López, Miguel Sampayo-Cordero, Javier Cortés, Antonio Llombart-Cussac

**Affiliations:** 1 Hospital Universitario 12 de Octubre, 28041 Madrid, Spain; 2 International Breast Cancer Center (IBCC), Pangaea Oncology, Quirón Group, 08022 Barcelona, Spain; 3 Medica Scientia Innovation Research (MEDSIR), 08005 Barcelona, Spain; 4 Instituto Valenciano de Oncología, 46009 Valencia, Spain; 5 Vall d’Hebron University Hospital, 08035 Barcelona, Spain; 6 Hospital Clínico San Carlos, 28040 Madrid, Spain; 7 Complejo Asistencial de Ávila, 05071 Ávila, Spain; 8 Hospital Universitario Virgen de la Victoria, 29010 Málaga, Spain; 9 University Hospital Reina Sofia, 14004 Córdoba, Spain; 10 Department of Medicine, Faculty of Biomedical and Health Sciences, Universidad Europea de Madrid, 28670 Madrid, Spain; 11 IOB Madrid, Institute of Oncology, Hospital Beata María Ana, 28007 Madrid, Spain; 12 Ribera Group, Oncology Department, Hospital Universitario Torrejón, 28850 Madrid, Spain; 13 Hospital Universitario Arnau de Vilanova, 46015 Valencia, Spain; 14 Translational Oncology Group, Department of Medicine, Facultad de Ciencias de la Salud, Universidad Cardenal Herrera-CEU, 46115 Alfara del Patriarca, Spain

**Keywords:** *BRCA* mutation, HR+/HER2− breast cancer, niraparib, advanced breast cancer, aromatase inhibitors

## Abstract

**Background:** Niraparib is an oral poly (adenosine diphosphate-ribose) polymerase inhibitor with promising activity for patients with advanced breast cancer harboring germline *BRCA*1/2 mutations. **Methods:** LUZERN (NCT04240106) was a multicenter, open-label, Simon’s two-stage, phase II clinical trial evaluating the efficacy and safety of niraparib with aromatase inhibitors (AIs) for patients with HR-positive/HER2-negative advanced breast cancer with either a germline *BRCA*1/2 mutation (cohort A) or germline *BRCA*1/2 wild-type and homologous recombination deficiency (exploratory cohort B). Eligible patients received ≤1 line of chemotherapy and 1–2 prior lines of endocrine therapy for advanced disease with secondary resistance to the last AI-based regimen. Patients received niraparib (300 mg or 200 mg) plus an AI. The primary endpoint was the clinical benefit rate (CBR) in cohort A. **Results:** Between June 2020 and November 2022, 14 patients were enrolled in cohort A (n = 6 for stage I, n = 8 for stage II) and no patients were enrolled in cohort B. One patient was excluded from the efficacy analysis due to no prior AI treatment. Nearly all patients (92.9%) previously received a cyclin-dependent kinase 4/6 inhibitor, but no patients had received prior platinum-based chemotherapy. Median follow-up was 16.7 months (range: 13.2–18.2). The CBR was 46.2% (95% CI: 19.2–74.9), meeting the primary endpoint. Median progression-free survival was 5.5 months (95% CI: 1.9–8.5), and median overall survival was 18.1 months (95% CI: 9.7–NE). The safety profile was consistent with the known toxicity of both drugs. **Conclusions:** Niraparib combined with an AI has encouraging antitumor activity and a manageable safety profile in patients with AI-resistant HR-positive/HER2-negative advanced breast cancer with germline *BRCA*1/2 mutations.

## Simple Summary

This study focuses on finding better treatments for women with advanced breast cancer who have certain genetic mutations. BRCA proteins are essential for repairing damaged DNA, and when these proteins are defective, it can lead to breast cancer. Women with *BRCA* mutations are at a higher risk of developing this cancer. The LUZERN study explored the combination of two drugs—niraparib, a PARP inhibitor, and aromatase inhibitors—to treat women with HR-positive/HER2-negative advanced breast cancer, particularly those with *BRCA* mutations or other DNA repair issues. The main goal was to see if this combination could provide a clinical benefit, meaning if it helped shrink the tumors or stabilize the disease for at least 24 weeks. This study found promising results, suggesting that this combination therapy might be an effective treatment for these patients, although more research is needed to confirm its benefits. The findings could help improve treatment strategies for patients with specific genetic profiles.

## 1. Introduction

Breast cancer remains the most frequently diagnosed malignancy among women worldwide and is a leading cause of cancer-related mortality. It is a heterogeneous disease comprising multiple subtypes, each characterized by distinct molecular profiles, clinical behaviors, and therapeutic responses [1]. Standard treatment strategies are tailored to the specific subtype and stage of the disease and may include surgery, radiation therapy, chemotherapy, endocrine therapy, targeted agents, and immunotherapy. Comprehensive strategies emphasizing prevention, early detection, screening, and treatment interventions are crucial [2].

Deoxyribonucleic acid (DNA) damage and deficiencies in repair mechanisms are central characteristics of cancer, with 5–10% of breast cancer patients harboring inherited DNA mutations [3]. *BRCA*1/2 gene mutations are the most well-known defects in DNA repair and have been estimated to account for around 2.5% of breast cancer cases and approximately 30% of cases with a positive family history of breast or ovarian cancer [3,4,5,6,7].

The BRCA proteins play a central role in DNA repair through the homologous recombination repair (HRR) pathway. BRCA1 forms a scaffold that organizes proteins at the DNA repair site, shifting repair towards HRR, whereas BRCA2 facilitates HRR through the recruitment of recombinase RAD51 [8]. However, *BRCA-*mutated cells are unable to repair double-stranded breaks through HRR, making them more vulnerable to rapid and uncontrolled growth.

*BRCA*1 mutation carriers are predisposed to triple-negative breast cancer (TNBC), whereas *BRCA*2 mutation carriers more frequently develop estrogen receptor–positive tumors [9,10].

Currently, cyclin-dependent kinase 4 and 6 inhibitor (CDK4/6i)-based endocrine therapy (ET) represents the standard of care in the first-line setting for patients with hormone receptor-positive/human epidermal growth factor receptor 2-negative (HR+/HER2−) advanced breast cancer, demonstrating significant improvements in both progression-free survival (PFS) and overall survival (OS) [11]. However, following disease progression on a CDK4/6i-containing regimen, single-agent ET typically offers limited clinical benefit, underscoring the importance of identifying actionable mutations to inform subsequent therapeutic strategies [12,13].

In this context, combining ET with targeted agents, such as inhibitors of the phosphatidylinositol 3-kinase (PI3K), mammalian target of rapamycin (mTOR), or protein kinase B (AKT) pathways (e.g., alpelisib, everolimus, or capivasertib, respectively), may enhance efficacy, particularly in patients harboring PIK3CA, AKT1, or phosphatase and tensin homolog (PTEN) alterations. Additionally, elacestrant, a selective estrogen receptor degrader, is a viable option for tumors with estrogen receptor 1 (ESR1) mutations. For patients with germline *BRCA*1/2 mutations, poly (ADP-ribose) polymerase (PARP) inhibitors may provide a targeted therapeutic alternative.

Breast cancer with a germline *BRCA*1/2 mutation (g*BRCA*m) is sensitive to DNA-damaging therapies, such as platinum therapies and poly (ADP-ribose) polymerase (PARP) inhibitors. Currently, two PARP inhibitors, olaparib and talazoparib, are approved in the United States and Europe as monotherapies for human epidermal growth factor 2 (HER2)-negative, g*BRCA*m, advanced breast cancer. This was based on two phase III clinical trials, OlympiAD and EMBRACA, where both olaparib and talazoparib, respectively, demonstrated a statistically significant improvement in progression-free survival (PFS) over standard-of-care chemotherapy for this patient population [14,15]. Interestingly, some breast cancer tumors also harbor homologous repair deficiencies (HRDs) that behave like *BRCA* mutations, making them potentially susceptible to DNA-damaging therapies [16,17,18,19,20,21].

Niraparib is a potent oral, selective PARP-1 and PARP-2 inhibitor that has shown promising clinical activity in patients with advanced ovarian [22] and breast cancer [23,24,25]. The phase III BRAVO trial compared the efficacy and safety of niraparib monotherapy to commonly used chemotherapy regimens for patients with g*BRCA*m and HER2-negative advanced breast cancer but was terminated prematurely due to the high rate of discontinuation in the control arm. However, with a median PFS of 4.1 months and an overall response rate (ORR) of 35%, the results suggest that niraparib could be a potential treatment option for this heavily pretreated patient population [25].

The LUZERN trial was a multicenter, open-label, phase II clinical trial that assessed the efficacy and safety of niraparib plus aromatase inhibitors (AIs) for patients with pretreated hormone receptor-positive/HER2-negative (HR[+]/HER2[−]) advanced breast cancer harboring a g*BRCA*m (cohort A) or who exhibited germline *BRCA*1/2 wild-type (g*BRCA*wt) and HRDs based on the Myriad myChoice^®^ CDx PLUS test developed by Myriad Genetic Laboratories, Inc., Salt Lake City, UT, USA) (exploratory cohort B).

## 2. Materials and Methods

### 2.1. Trial Design

LUZERN was a multicenter, open-label, single-arm, two-cohort, Simon’s two-stage phase II clinical trial that assessed the efficacy and safety of niraparib plus an AI for HR[+]/HER2[−] advanced breast cancer patients with secondary resistance to the last AI-based regimen and harboring a g*BRCA*m (cohort A) or g*BRCA*wt and HRD based on the Myriad myChoice^®^ CDx PLUS test (exploratory cohort B).

The trial used a Simon’s two-stage minimax design where patients were recruited in two study cohorts: cohort A, g*BRCA*m (N = 6 for stage I; N = 8 for stage II), and exploratory cohort B, g*BRCA*wt and HRD (N = 9 for stage II). An interim analysis was planned when the first six patients had enrolled. At least one patient in stage I of cohort A had to have a clinical benefit to continue to stage II, which included both cohorts.

Enrolled patients were to receive niraparib (300 mg for ≥77 kg and ≥150,000 platelets/µL; 200 mg for <77 kg or <150,000 platelets/µL) orally once daily, flat fixed, and continuously in a 28-day cycle in combination with an AI that was identical to the last AI-containing regimen in which the patient had progressed to the advanced setting. Treatment continued until disease progression, unacceptable toxicity, death, or discontinuation from the study for any other reason.

### 2.2. Patients

Eligible patients included men and pre-/post-menopausal women aged 18 or older with HR[+]/HER2[–] advanced breast cancer who were not candidates for curative intent and had a deficiency in the HRR pathway. Participants had evidence of either measurable or evaluable disease as per the Response Evaluation Criteria in Solid Tumors (RECIST) version 1.1. Participants included in cohort A exhibited a g*BRCA*m, and exploratory cohort B patients exhibited g*BRCA*wt and an HRD based on the Myriad myChoice^®^ CDx PLUS test.

Eligible patients received 1–2 previous endocrine-based therapy regimens for advanced disease and confirmed progression on the last AI-containing regimen (not necessarily in the treatment line immediately prior to treatment) with secondary endocrine resistance criteria (disease progression on adjuvant treatment after at least two years of ongoing therapy and within 12 months following treatment interruption or during treatment for advanced disease after at least six months of ongoing therapy and within six weeks following treatment interruption). Patients could have received no more than one chemotherapy regimen in the advanced setting. Prior platinum compound-based therapy was allowed if the disease-free interval was 12 months or longer. Pre-menopausal women had to be treated with luteinizing hormone and relapsing hormone analogs for at least 28 days prior to study entry. Key exclusion criteria included patients with HER2[+] disease and previous treatment with PARP inhibitors for advanced disease. The full inclusion and exclusion criteria are provided in the Supplementary Material File S1.

### 2.3. Trial Oversight

This trial was conducted following the guidance of the International Conference on Harmonization and the ethical principles outlined in the Declaration of Helsinki. It was approved by the ethics review committees of each participating institution. All patients provided written informed consent. The trial registration number (clinicaltrial.gov) is NCT04240106.

### 2.4. Endpoints and Assessments

The primary endpoint of the LUZERN study was the clinical benefit rate (CBR), defined as the percentage of patients who experienced a complete response, partial response, or stable disease for at least 24 weeks. The CBR was assessed locally by the Investigator with Response Evaluation Criteria in Solid Tumors (RECIST) v.1.1 criteria.

Secondary efficacy endpoints included the PFS, ORR, time to response (TTR), duration of response (DoR), overall survival (OS), maximum tumor reduction, and safety and efficacy. The PFS, ORR, TTR, DoR, and maximum tumor reduction were assessed locally by the Investigator using RECIST v.1.1 criteria. Tumor assessments were carried out every eight weeks from the first study dose for the first six months, and then every 12 weeks thereafter until the end of the study. Safety was evaluated throughout the study and the occurrence and maximum grade of side effects were recorded and assessed according to the National Cancer Institute (NCI) Common Terminology Criteria for Adverse Events (CTCAE) v.5.0.

### 2.5. Statistical Analysis

The efficacy analysis included the per-protocol patients, excluding one patient who did not previously receive an AI. The safety analysis included all patients enrolled in this study.

A sample size of 14 patients for cohort A and 9 patients for exploratory cohort B was needed to attain 80% power at the nominal level of one-sided alphas of 0.025 and 0.1, respectively. Given that this was an exploratory study designed to describe the level of activity in a heavily pretreated population with secondary resistance to the last AI-based regimen, the primary analysis tested the hypothesis that the CBR estimated in cohort A in the final analysis was less than or equal to 5%. The CBR was calculated with the stochastic ordering of the UMVUE method. Three or more patients needed to have a clinical benefit at either the interim or final analysis for the primary endpoint to be met.

The primary analysis calculated the CBR according to RECIST v.1.1, along with 95% Pearson–Clopper confidence intervals (CIs) and *p*-values under the exact binomial test. For binary endpoints, the number and proportion of patients with responses, and the 95% Pearson–Clopper CI, were calculated. Kaplan–Meier was applied to all time and event endpoints. The number and proportion of events, median survival, and survival rates, with corresponding 95% CIs, were calculated. All analyses were conducted in SAS® v.9.4 and R v.4.2.2.

## 3. Results

### 3.1. Patients

**Figure 1:**
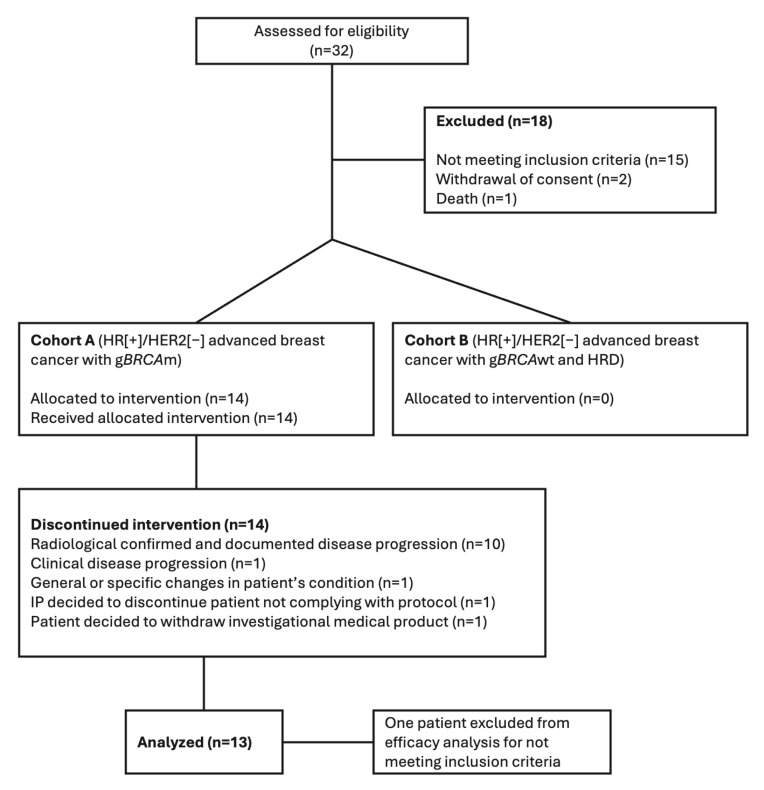
Patient flow chart. Abbreviations: g*BRCA*m: germline *BRCA* mutation; g*BRCA*wt: germline *BRCA* wild-type; HRD: homologous recombination deficiency; HR+/HER2−: hormone receptor-positive/HER2-negative.

From 15 June 2020 to 17 November 2022, 32 patients were assessed for eligibility, and 14 patients were enrolled in this study (Figure 1). All 14 patients included were part of cohort A, with six patients in stage I and an additional eight patients in stage II. Seven of the thirty-two patients screened were for exploratory cohort B; however, none were included due to not fulfilling selection criteria (five patients), withdrawal of consent (one patient), and death (one patient).

Patient demographics are described in Table 1. The median age was 46 years (range: 32–83), with 57.1% of patients having an Eastern Cooperative Oncology Group (ECOG) score of 0 and 71.4% of patients having visceral involvement. The median number of lines of therapy for advanced disease was two (range: 0–7). Nearly all (92.9%) had previous treatment with a cyclin-dependent kinase (CDK) 4/6 inhibitor. Five patients had one previous line of chemotherapy for advanced disease, but none were platinum-based.

Two patients in stage I of cohort A withdrew consent and were deemed non-evaluable, as detailed in Figure 1.

### 3.2. Efficacy Analysis

At the data cutoff (14 November 2023) for final analysis, no patient remained on treatment, with progressive disease (78.6%) as the main reason for treatment discontinuation. The median duration of follow-up was 16.7 months (range: 13.2–18.2). One patient was excluded from the efficacy analysis due to no prior AI treatment.

**Figure 2:**
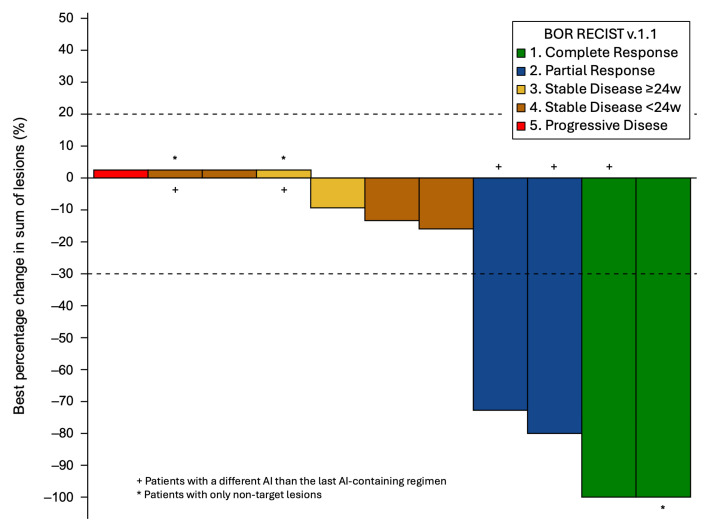
Waterfall plot of the best change from baseline in tumor lesions according to the best objective response. Abbreviations: AI: aromatase inhibitor; w: weeks.

**Figure 3:**
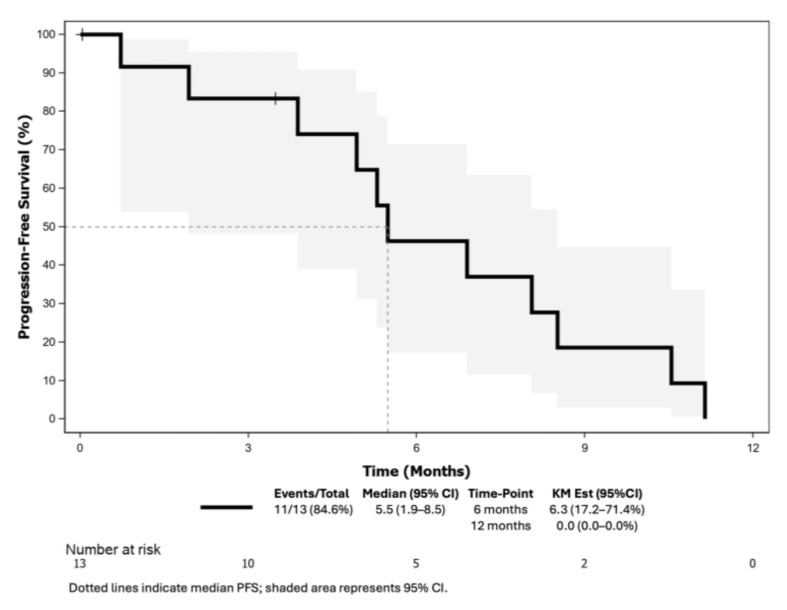
Progression-free survival of patients included in the LUZERN trial. Abbreviations: KM Est: Kaplan–Meier estimated; 95% CI: 95% confidence interval.

**Figure 4:**
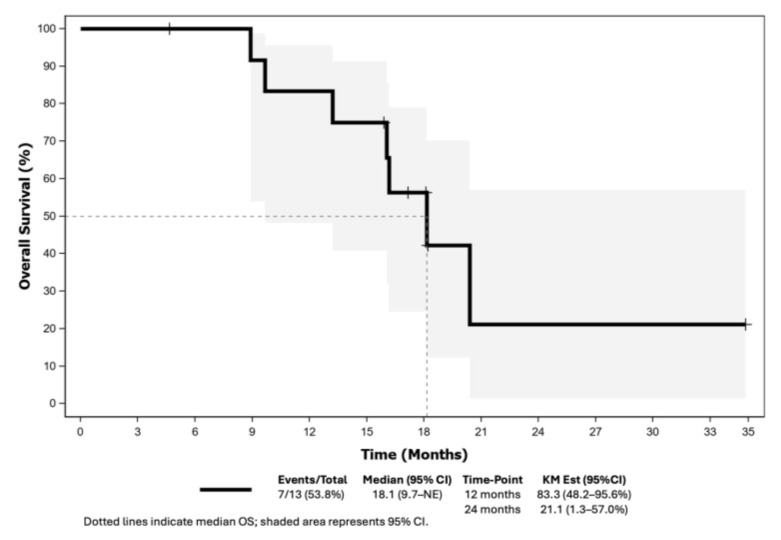
Overall survival of patients included in the LUZERN trial. Abbreviations: KM Est: Kaplan–Meier estimated; 95% CI: 95% confidence interval.

The CBR for cohort A was 46.2% (95% CI: 19.2–74.9), meeting the primary endpoint (Table 2). The ORR was 30.8% (95% CI: 9.1–61.4), with two patients with complete responses and two patients with a partial response (Figure 2). The median TTR and DoR were 1.7 months (95% CI: 1.7–NE) and 5.6 months (95% CI: 3.3–8.8), respectively. With eleven (84.6%) and seven (53.5%) PFS and OS events, the median PFS was 5.5 months (95% CI: 1.9–8.5) (Figure 3) and the median OS was 18.1 months (95% CI: 9.7–NE) (Figure 4).

#### Efficacy Analysis Based on Previous AIs

Despite the inclusion criteria indicating that patients must have had disease progression on the last AI-containing regimen, five of the thirteen patients received a different AI, and eight received the same AI as in the prior line.

Among the patients treated with a different AI from the last AI-containing regimen (N = 5), the CBR was 80.0% (95% CI: 28.4–99.5), the ORR was 60.0% (95% CI: 14.7–94.7) (Table 2), and the median PFS was 8.0 months (95% CI: 0.7–11.1). For patients with the same AI as in the last AI-containing regimen (N = 8), the CBR was 25.0% (95% CI: 3.2–65.1), the ORR was 12.5% (95% CI: 0.3–52.7) (Table 2), and the median PFS was 5.5 months (95% CI: 1.9–8.5).

### 3.3. Safety

The safety results are summarized in Table 3, which includes all patients who received at least one dose of treatment (100.0%). Thirteen patients (92.9%) reported at least one treatment-emergent adverse event (TEAE). The most common TEAEs of any grade were fatigue (71.4%), nausea (57.1%), neutropenia (35.7%), vomiting (28.6%), and anemia (28.6%). The reported grade 3 TEAEs observed were anemia (14.3%), neutropenia (7.1%), thrombocytopenia (7.1%), nausea (7.1%), vomiting (7.1%), COVID-19 pneumonia (7.1%), and pericardial effusion (7.1%).

Eleven patients (78.6%) had dose interruptions, with toxicity (42.9%) as the most common reason. Seven patients (50.0%) required a dose reduction. One patient (7.1%) experienced grade 2 anxiety, which led to permanent discontinuation.

Three patients (21.4%) had serious TEAEs, which included COVID-19 pneumonia, pseudomonal bacteremia, and a pericardial effusion, but all were unrelated to the study treatment. No grade 4 TEAEs, treatment-related deaths, or cases of myelodysplastic syndrome or acute myelogenous leukemia were reported.

## 4. Discussion

In this open-label, single-arm, phase II study, the combination of niraparib with an AI showed encouraging antitumor activity and a manageable safety profile for patients with AI-resistant HR[+]/HER2[−] advanced breast cancer. This study met its primary endpoint, with a CBR of 46.2% (95% CI: 19.2–74.9). The ORR was 30.8% (95% CI: 9.1–61.4), with two patients with complete responses and two patients with partial responses.

While CDK4/6 inhibitors are the standard first-line treatment for patients with HR[+]/HER2[−], the best treatment after progression for g*BRCA*m patients remains unclear. Several studies have demonstrated the clinical activity of PARP inhibitor monotherapy for patients with advanced breast cancers that harbor g*BRCA*m. The OlympiAD trial was an open-label, randomized, phase III trial that determined that olaparib was superior, in terms of PFS, to the physician’s choice single-agent chemotherapy in patients with g*BRCA*m, HER2[−] advanced breast cancer [14,26]. Similarly, talazoparib was found to have a significantly longer PFS versus single-agent chemotherapy in patients with g*BRCA*m advanced breast cancer in the randomized phase III EMBRACA trial [15,27]. Accordingly, both treatments are approved for patients with HR[+] advanced breast cancer who have progressed on or after prior endocrine therapy or are considered unsuitable for endocrine therapy.

Interestingly, the OlympiAD and EMBRACA trials did not include patients who were previously treated with CDK4/6 inhibitors as they were not approved at the start of the trials, and it is unclear if previous treatment with CDK4/6 inhibitors could negatively impact the efficacy of PARP inhibitors. However, in clinical practice, olaparib and talazoparib are usually used in patients progressing to prior CDK4/6 inhibitor-based therapy. In contrast, nearly all the patients (92.9%) included in LUZERN were previously treated with a CDK4/6 inhibitor. Despite this fact, the antitumor activity of niraparib remains promising. On the other hand, real-world and retrospective studies have indicated that CDK4/6 inhibitor treatment had worse outcomes for patients with HR[+] breast cancer harboring g*BRCA*m [28,29]. However, to date, no studies have compared PARP inhibitors head-to-head with CDK4/6 inhibitors. Therefore, further research is needed to optimize sequencing for this patient population.

Additionally, it is important to note that neither trial combined PARP and endocrine therapy; therefore, olaparib and talazoparib are approved as monotherapies even for HR[+] advanced breast cancer, and it is unknown if the addition of endocrine therapy to PARP inhibitors could have a synergistic effect in these patients. In our opinion, the combination of an AI or other endocrine therapy with either talazoparib or olaparib could be a potential treatment strategy to explore in this patient population, with the aim of increasing the efficacy of PARP inhibitors. Recently, the DOLAF trial explored the combination of olaparib, durvalumab, and fulvestrant, demonstrating promising results [30].

Niraparib is approved as a maintenance treatment for platinum-sensitive recurrent advanced ovarian cancer that previously responded to platinum chemotherapy. Additionally, the BRAVO trial confirmed its activity in patients with advanced breast cancer and g*BRCA*m [25]. Other studies also demonstrated niraparib’s promising clinical activity as neoadjuvant treatment for localized HER2[−], g*BRCA*m breast cancer and in triple-negative breast cancer, irrespective of g*BRCA*m status [23,24]. The safety profile of niraparib was in line with previous studies, with no new safety signals [22,23,25]. The most common TEAEs in LUZERN were nausea, fatigue, and neutropenia.

LUZERN was a non-randomized phase II clinical trial. A main limitation of LUZERN was the sample size. While this was a proof-of-concept study, the number of patients was small, limiting the ability to draw any large conclusions. This study also had protocol deviations in five patients, which affects the interpretation of the results but did reveal a potential benefit of switching the aromatase inhibitor, which should be further explored. Additionally, this study was limited in that no patients were enrolled in exploratory cohort B. HRD incidence, as determined by the Myriad myChoice^®^ HRD Plus CDx test in g*BRCA*wt advanced breast cancer, appears to be infrequent; therefore, it can be difficult to carry out formal phase II or III studies for these molecular alterations. Given the low incidence, it is important to identify patients who could benefit from PARP inhibition beyond g*BRCA*m [18]. Developing large randomized clinical trials remains a challenge for mutations with low incidence rates. Future studies should explore alternative treatment combinations, including other DNA damage response inhibitors or targeted therapies, to optimize outcomes in this patient population. Additionally, adaptive trial designs may be useful to accommodate low-prevalence genetic subgroups.

## 5. Conclusions

In conclusion, the LUZERN trial provides further evidence of the clinical activity and safety profile of niraparib, warranting further investigation of niraparib and AIs for patients with g*BRCA*m advanced breast cancer.

## Figures and Tables

**Table 1:** Patient baseline characteristics.

Baseline Characteristics; N = 14	n (%)
Age, Median (min; max) years	46 (32; 83)
ECOG	
0	8 (57.1%)
1	6 (42.9%)
Visceral involvement	
No	4 (28.6%)
Yes	10 (71.4%)
Number of metastatic sites	
1	3 (21.4%)
2	5 (35.7%)
≥3	6 (42.9%)
Previous treatment with targeted therapy for advanced disease	
CDK4/6 inhibitor	13 (92.9%)
mTOR inhibitor	1 (7.1%)
Previous lines of chemotherapy for advanced disease	
0	9 (64.3%)
1	5 (35.7%)
Previous lines of systemic therapy for advanced disease	
0	1 (7.1%)
1	6 (42.9%)
2	3 (21.4%)
≥3	4 (28.6%)
Patients treated with an AI different from the last AI-containing regimen	
No	8 (57.1%)
Yes	6 (42.9%)

Abbreviations: AI: aromatase inhibitor; CDK4/6: cyclin-dependent kinase; ECOG: Eastern Cooperative Oncology Group; mTOR: mammalian target of rapamycin; N: number of patients; n (%): number of patients (percentage of patients).

**Table 2:** Overall response of LUZERN trial participants.

	Different AI (N = 5)	Same AI (N = 8)	Overall (N = 13)
Clinical Benefit Rate (CBR)			
No	1 (20.0%)	6 (75.0%)	7 (53.8%)
Yes	4 (80.0%)	2 (25.0%)	6 (46.2%)
95% CI	(28.4%; 99.5%)	(3.2%; 65.1%)	(19.2%; 74.9%)
Best Overall Response (BOR)			
CR	1 (20.0%)	1 (12.5%)	2 (15.4%)
PR	2 (40.0%)	0 (0.0%)	2 (15.4%)
SD ≥ 24 w	1 (20.0%)	1 (12.5%)	2 (15.4%)
SD < 24 w	0 (0.0%)	4 (50.0%)	4 (30.8%)
PD	0 (0.0%)	1 (12.5%)	1 (7.7%)
NE	1 (20.0%)	1 (12.5%)	2 (15.4%)
Objective Response Rate (ORR)			
No	2 (40.0%)	7 (87.5%)	9 (69.2%)
Yes	3 (60.0%)	1 (12.5%)	4 (30.8%)
95% CI	(14.7%; 94.7%)	(0.3%; 52.7%)	(9.1%; 61.4%)

Abbreviations: AI: aromatase inhibitor; CR: complete response; NE: not evaluable; PD: progressive disease; PR: partial response; SD: stable disease; 95% CI: 95% confidence interval.

**Table 3:** TEAEs and related TEAEs in >10% of patients.

Overall (N = 14)	TEAEs		Related TEAEs	
n (%)	Any	G3	Any	G3
Any	13 (92.9%)	6 (42.9%)	10 (71.4%)	4 (28.6%)
Hematologic				
Neutropenia	7 (50.0%)	1 (7.1%)	5 (35.7%)	1 (7.1%)
Anemia	7 (50.0%)	2 (14.3%)	3 (21.4%)	1 (7.1%)
Thrombocytopenia	4 (28.6%)	1 (7.1%)	2 (14.3%)	1 (7.1%)
Leukopenia	2 (14.3%)	0 (0.0%)	1 (7.1%)	0 (0.0%)
Non-hematologic				
Fatigue	10 (71.4%)	0 (0.0%)	5 (35.7%)	0 (0.0%)
Nausea	8 (57.1%)	1 (7.1%)	7 (50.0%)	1 (7.1%)
Vomiting	4 (28.6%)	1 (7.1%)	4 (28.6%)	1 (7.1%)
Abdominal discomfort	2 (14.1%)	0 (0.0%)	1 (7.1%)	0 (0.0%)
Constipation	2 (14.1%)	0 (0.0%)	1 (7.1%)	0 (0.0%)
Limb discomfort	2 (14.1%)	0 (0.0%)	0 (0.0%)	0 (0.0%)
Musculoskeletal chest pain	2 (14.1%)	0 (0.0%)	0 (0.0%)	0 (0.0%)
Palpitations	2 (14.1%)	0 (0.0%)	0 (0.0%)	0 (0.0%)

Abbreviations: TEAEs: treatment-emergent adverse events; n (%): number of patients (percentage of patients).

## Data Availability

Data collected within this study will be made available to researchers after contacting the corresponding author and upon revision and approval based on scientific merit by the LUZERN trial management group (which includes a qualified statistician) of a detailed proposal for their use. The data required for the approved, specified purposes, the trial protocol, and the statistical analysis plan will be provided after the completion of a data sharing agreement that will be set up by the study sponsor, beginning 1 month and ending 5 years after article publication. All data provided are anonymized to respect the privacy of patients who have participated in the trial in line with applicable laws and regulations. The estimated timeframe for response will be within 30 days. Please address requests for data to the corresponding author.

## Supplementary Materials

The following supporting information can be downloaded at: https://www.mdpi.com/article/10.3390/cancers17111744/s1, File S1: Patient enrollment criteria.

## Abbreviations

The following abbreviations are used in this manuscript:

AI	Aromatase inhibitor
BRCA	Breast cancer gene
CBR	Clinical benefit rate
CDK	Cyclin-dependent kinase
CI	Confidence interval
CR	Complete response
CTCAE	Common Terminology Criteria for Adverse Event
DNA	Deoxyribonucleic acid
DoR	Duration of response
ECOG	Eastern Cooperative Oncology Group
g*BRCA*m	Germline *BRCA*1/2 mutation
g*BRCA*wt	Germline *BRCA*1/2 wild type
HER2	Human epidermal growth factor 2
HR	Hormonal receptor
HRD	Homologous repair deficiencies
HRR	Homologous recombination repair
KM Est	Kaplan–Meier estimated
mTOR	Mammalian target of rapamycin
N	Number of patients
NE	Not evaluable
NCI	National Cancer Institute
OS	Overall survival
ORR	Overall response rate
PARP	Poly (adenosine diphosphate-ribose) polymerase
PD	Progressive disease
PFS	Progression-free survival
PR	Partial response
RAD51	Radiation-sensitive gene 51
RECIST	Response Evaluation Criteria in Solid Tumors
SD	Stable disease
TEAE	Treatment-emergent adverse event
TTR	Time to response
W	Weeks
n (%)	Number of patients (percentage of patients)
