# Supplementary material for: Niraparib Plus Aromatase Inhibitors for Hormone Receptor-Positive/HER2-Negative Advanced Breast Cancer with a Germline BRCA Mutation
Source: Cancers (Basel). 2025 May 22;17(11):1744. doi: 10.3390/cancers17111744 (PMC12153925; doi:10.3390/cancers17111744)
Supplement: Supplementary file 1 [file cancers-17-01744-s001.zip › cancers-3610879-supplementary.pdf]

Niraparib Plus Aromatase Inhibitors for Hormone Receptor-Positive/HER2-Negative Advanced Breast Cancer with a Germline *BRCA* Mutation

Supplementary Materials and Methods

Supplementary Methods File S1

Patient enrollment criteria

Inclusion criteria

Patient eligibility was reviewed and documented by a suitable member of the study team before the patients were enrolled in the study.

Patients met all the following inclusion criteria to be enrolled in the study:

1. Patients were informed about the nature of the study, including the exploratory sub-study, and agreed to participate and signed the informed consent prior to participation in any study-related activities.
2. Male or female patients ≥ 18 years of age.
3. Eastern Cooperative Oncology Group (ECOG) performance status of 0 or 1 which the Investigator believed was stable at the time of screening.
4. Life expectancy ≥16 weeks.
5. Patients had a histologically and/or cytologically confirmed diagnosis of breast cancer.
6. Patients had radiologic evidence of inoperable locally recurrent or metastatic breast cancer (MBC) and were not candidates for curative intent.
7. Patients had human epidermal growth factor receptor 2 (HER2)-negative breast cancer (based on most recently analyzed biopsy) defined as a negative *in situ* hybridization (ISH) test or an immunohistochemistry (IHC) status of 0, 1+, or 2+ (if IHC 2+, a negative ISH test is required) by local laboratory testing. *Note: HER2 tests which preceded the ICF signature were accepted, as long as the results were documented and captured in the medical record during the pre-screening period.*
8. Patients had hormone receptor (HR)-positive breast cancer (based on most recently analyzed biopsy) defined as estrogen receptor (ER) and/or progesterone receptor (PgR) with ≥10% of tumor cells positive for ER and/or PgR by IHC irrespective of staining intensity. *Note: HR tests which preceded the ICF signature were accepted, as long as the results were documented and captured in the medical record during the pre-screening period*.
9. [Cohort A]: Patients with documented germinal mutation in *BRCA1* or *BRCA2* genes that was predicted to be deleterious or suspected deleterious (known or predicted to be detrimental/lead to loss of function). Patients with germinal mutations in *BRCA1 or BRCA2* genes (g*BRCA*m) that were considered to be non-detrimental (e.g., “Variants of uncertain clinical significance” or “Variant of unknown significance” or “Variant, favor polymorphism” or “benign polymorphism,” etc.) were not eligible for the study. Germinal *BRCA1/2* results preceding the ICF signature were accepted, as long as the

results were documented and captured in the medical record during the pre-screening period.

10. [Exploratory cohort B]: Patients with either germinal *BRCA1/2* wild-type (g*BRCA*wt) or g*BRCA*m that was considered to be non-detrimental (e.g., "Variants of uncertain clinical significance" or "Variant of unknown significance" or "Variant, favor polymorphism" or "benign polymorphism," etc.) and homologous recombination deficiency (HRD) based on the Myriad myChoice® CDx PLUS test. HRD status was centrally confirmed both on the most recent tumor tissue since last progression (from either metastasis or primary tumor) and blood samples. Patients with a Myriad myChoice® CDx PLUS score of ≥ 25 or greater were considered to have an abnormal HRR pathway and defined as HRD.

Note 1: Germinal BRCA assays carried out before the pre-screening were accepted, as long as the results were documented and captured in the medical record during the pre-screening period.

Note 2: Subjects for whom the most recent tumor biopsy since last progression could not be obtained (e.g., inaccessible tumor or subject safety concern) could submit archival pathological material from either metastatic or primary sites, but the most recent tumor biopsy from the patient needed to be obtained when available.

11. [Exploratory cohort B]: Willingness and ability to provide additional six formalin-fixed paraffin-embedded (FFPE) tissue slides from the most recent tumor tissue since last progression (from either metastasis or primary tumor) to centrally perform the RAD51 assay. It was strongly recommended to obtain six consecutive sections from the same tissue block used for the determination of HRD status.

Note 1: Subjects for whom the most recent tumor biopsy since last progression could not be obtained (e.g., inaccessible tumor or subject safety concern) could submit archival pathological material from either metastatic or primary sites, but the most recent tumor biopsy from the patient needed to be obtained when available.

Note 2: Results of this exploratory sub-study were not to be used to confirm patient's eligibility.

12. At least one and up to two prior lines of endocrine therapy (AIs or fulvestrant) for treatment of locally recurrent and/or metastatic disease (except for patients progressing in the neoadjuvant or adjuvant setting).

13. Confirmed disease progression while on the last AI-containing regimen (not necessarily in the treatment line immediately prior to study entry) with secondary endocrine resistance criteria defined as:

- Progression on adjuvant AI-based regimen, confirmed after at least 2 years of ongoing therapy and within 12 months following adjuvant treatment interruption; OR,
- Progression to at least one AI-based regimen for treatment of locally recurrent and/or metastatic disease after having achieved clinical benefit (at least 24 weeks on treatment).

Confirmation of progression must be within 6 weeks after the end of treatment for locally recurrent and/or metastatic disease.

14. Patients could not have progressed on more than one chemotherapy regimens in the metastatic setting.
15. The following was not to be counted as a prior line of cytotoxic chemotherapy:

- Prior hormonal therapy and non-hormonal targeted therapy.
- Targeted and biologic therapies.
- The patient could receive a stable dose of bisphosphonates or denosumab for bone metastases, before and during the study as long as this was started at least 5 days prior to study treatment.

16. Prior carboplatin- or other platinum compound-based therapy was allowed if have been administered in one of the following settings:

- Disease-free interval > 12 months from date of completion of neoadjuvant or adjuvant treatment.
- As potentially curative treatment for a prior non-breast cancer with no evidence of disease for ≥ 5 years.

17. Patients had evaluable or measurable disease according to RECIST criteria v1.1. Patients with bone-only metastases are eligible.
18. Willingness and ability to provide the most recent tumor biopsy since last progression from either metastatic or primary tissues both at the time of the inclusion and at disease progression or study termination in order to perform exploratory studies. If not feasible, patient eligibility was evaluated by a Sponsor's qualified designee.

Note: Subjects for whom tumor biopsy could not be obtained (e.g., inaccessible tumor or subject safety concern) could submit archival pathological material from either metastatic or primary sites, but the most recent tumor biopsy from the patient needed to be obtained when available at both timepoints.

19. Patients agreed to provide blood samples at the time of study inclusion, every three cycles of treatment (C4D1, C7D1, C10D1, …), and upon disease progression or study termination in order to perform exploratory studies.
20. Adequate hematologic and organ function within 28 days before the first study treatment on Cycle 1 Day 1, defined by the following:

- Hematological: White blood cell (WBC) count >3.0 x 10^9/L, absolute neutrophil count (ANC) ≥1.5 x 10^9/L, platelet count ≥100.0 x10^9/L, and hemoglobin ≥9 g/dL with no blood transfusion in the past 35 days;
- Hepatic: bilirubin ≤ 1.5 times the upper limit of normal (x ULN) (≤2.0 in patients with known Gilbert's syndrome) or direct bilirubin ≤ 1 x ULN; alkaline phosphatase (ALP), Aspartate aminotransferase (AST)/Serum Glutamic Oxaloacetic Transaminase (SGOT), and Alanine aminotransferase (ALT)/Serum Glutamic Pyruvate Transaminase (SGPT) ≤

2.5 x institutional ULN unless liver metastases are present, in which case they must be ≤ 5 x ULN;

- Renal: Serum creatinine ≤ 1.5 x ULN or calculated creatinine clearance ≥ 30 mL/min using the Cockcroft-Gault equation.

21. Female patients of childbearing potential had a negative serum pregnancy test within 3 days prior to study treatment and agreed to abstain from activities that could result in pregnancy from screening through 6 months after the last dose of study treatment, or patients of non-childbearing potential, where non-childbearing potential is defined as follows (by other than medical reasons):

- ≥45 years of age and has not had menses for >1 year;
- Patients who were amenorrhoeic for <2 years without history of a hysterectomy and oophorectomy must have levels of Luteinizing hormone (LH) and Follicle stimulating hormone (FSH) in the post-menopausal range for women <50;
- Post-hysterectomy, post-bilateral oophorectomy, or post-tubal ligation: documented hysterectomy or oophorectomy was confirmed with medical records of the actual procedure or confirmed by an ultrasound. Tubal ligation was confirmed with medical records of the actual procedure, otherwise the patient was willing to use 2 adequate barrier methods throughout the study, starting with the screening visit through 6 months after the last dose of study treatment. Information must be captured appropriately within the site's source documents.

22. Female patients agreed not to breastfeed during the study and for 1 month after the last dose of study treatment.
23. Male patients whose partners were women of childbearing potential used a condom during niraparib therapy and for 3 months after receiving the last dose of niraparib and agreed to abstain from activities that could result in pregnancy. In addition, men did not donate sperm during niraparib therapy and for 3 months after receiving the last dose of niraparib.

Exclusion criteria

Any patient meeting the following criteria was excluded from the study:

1. HER2-positive disease based on local laboratory results (performed by IHC/*in situ* hybridization test) or unknown HER2 status.
2. Patients that were candidates for a local treatment with a radical intention.
3. Patients that had previously received any PARP inhibitor (PARPi), including niraparib, in metastatic setting.

Note: Patients treated with PARPi on (neo)adjuvant regimen with disease-free interval greater than 24 months following treatment interruption were eligible.

4. Patients could not be simultaneously enrolled in any interventional clinical trial and could not have received investigational therapy ≤ 4 weeks, or within a time interval less than at least 5 half-lives of the investigational agent, whichever is shorter, prior initiating protocol therapy.
5. Patients who had radiation therapy encompassing >20% of the bone marrow within 2 weeks prior to start of treatment, excepting for palliative radiation therapy to a small field >1 week prior to Day 1 of study.
6. Patients with visceral crisis who required chemotherapy.
7. Patients could not have had a known hypersensitivity to niraparib components or excipients.
8. Patients could not have received a transfusion (platelets or red blood cells) ≤ 4 weeks prior to initiating protocol therapy.
9. Patients could not have received colony stimulating factors (e.g., Granulocyte colony-stimulating factor [G-CSF], granulocyte macrophage colony stimulating factor, or recombinant erythropoietin) within 4 weeks prior initiating protocol therapy.
10. Patients could not have had any known Grade 3 or 4 anemia, neutropenia or thrombocytopenia due to prior chemotherapy in adjuvant setting or CDK4/6 inhibitors that persisted > 4 weeks and was related to the most recent treatment.
11. Patients could not have had any known history of Myelodysplastic syndrome (MDS) or Acute myeloid leukemia (AML).
12. Patients could not have had a serious, uncontrolled medical disorder, nonmalignant systemic disease, or active, uncontrolled infection. Examples include, but are not limited to, uncontrolled ventricular arrhythmia, recent (≤ 90 days) myocardial infarction, uncontrolled major seizure disorder, unstable spinal cord compression, superior vena cava syndrome, extensive interstitial bilateral lung disease on High Resolution Computed Tomography (HRCT) scan, any psychiatric disorder that prohibits obtaining informed consent, severe immunodeficiency disorders (i.e. human immunodeficiency virus [HIV] infection) or active hepatitis (i.e. Hepatitis B or C).
13. Patients could not have had diagnosis, detection, or treatment of another type of cancer ≤ 2 years prior to initiating protocol therapy (except basal or squamous cell carcinoma of the skin, cervical carcinoma *in situ* and ductal carcinoma *in situ* [DCIS] definitively treated).
14. Patients with symptomatic uncontrolled brain metastases or leptomeningeal metastases. A scan to confirm the absence of brain metastases was not required. Patients with spinal cord compression unless considered to have received definitive treatment for this condition and have evidence of clinically stable disease (SD) for 35 days.
15. Previous allogenic bone marrow transplant or double umbilical cord blood transplantation (dUCBT).

16. Patients who were unable to swallow orally administered medication.
17. Patients with gastrointestinal disorders likely to interfere with absorption of the study medication.
18. Chronic daily treatment with corticosteroids with a dose of ≥ 10 mg/day methylprednisolone equivalent (excluding inhaled steroids), except for prophylaxis use.
19. Female patients who were pregnant or breastfeeding, or adults of reproductive potential who were not using effective birth control methods.
20. Patients unwilling to or unable to comply with the protocol for the duration of the study including undergoing treatment and scheduled visits and examinations.
